# Supplementary material for: A Randomized, Crossover Study of the Acute Cognitive and Cerebral Blood Flow Effects of Phenolic, Nitrate and Botanical Beverages in Young, Healthy Humans
Source: Nutrients. 2020 Jul 28;12(8):2254. doi: 10.3390/nu12082254 (PMC7468953; doi:10.3390/nu12082254)
Supplement: Supplementary file 1 [file nutrients-12-02254-s001.pdf]

Supplemental Table 1. Cognitive tasks completed at each assessment in order of presentation

Task	Descriptor	Scoring
Word presentation	A series of words is displayed on the screen, one word at a time. In this case, 15 words were presented with a display time of 1 second and inter-stimulus interval of 1 second.	-
Immediate word recall	Participants are instructed to write down the words that were presented. In this case, 60 seconds were given to complete the task.	Number correct and number of errors
Picture presentation	A series of photographic images are displayed on the screen, one at a time. In this case, 15 images were presented with a display time of 2 seconds and an inter-stimulus interval of 1 second.	-
Serial 3 subtractions	Participants are required to count backwards in threes from a given number as quickly and as accurately as possible using the computer keyboard linear number keys to enter each response. A random starting number between 800 and 999 is presented on the computer screen, which is cleared by the entry of the first response. The duration of this task is 2 minutes	
Serial 7 subtractions	As above, counting backwards in sevens.	
Rapid visual information processing	A continuous series of single digits are presented in the center of the screen at the rate of 100 per minute. Participants are required to make a response when three consecutive	Accuracy (%), reaction time for the correct

	odd or three consecutive even digits are displayed. The duration of this task is 5 minutes, with 8 correct target strings presented in each minute.	responses (ms) and false alarms (number)
Stroop task	In this computerized version of the classic task, words describing one of four colors (‘RED’, ‘YELLOW’, ‘GREEN’, and ‘BLUE’) are presented in different coloured fonts in the centre of the computer screen. The participant presses one of four coloured response buttons in order to identify the font colour (e.g. if the word ‘GREEN’ was presented in a blue font, the correct response would be to respond with the blue button). The presented words are either ‘congruent’ (word and font were the same colour) or ‘incongruent’ (word and font were different colours) and are presented in a random order.	Accuracy (%), reaction time for the correct responses (ms)
Peg and ball task	In this computerized version of the executive function task two configurations of three coloured balls (blue, green, and red) on three pegs that each hold a maximum of three balls are presented on screen. The participants have to rearrange the balls, moving one ball at a time, from the starting configuration so that they match the position of the balls in the goal configuration. Participants randomly complete five trials at each level, which can be solved in 3, 4, and 5 moves respectively.	Each trial generates scores for planning times prior to moving (ms), time to complete (ms) and errors (number).

Delayed word recall	Participants are instructed to write down the words that were presented to them at the beginning of the assessment. In this case, 60 seconds were given to complete the task	Number correct and number of errors
Delayed word recognition	All target words that were shown during Word presentation plus an equal number of decoys are displayed on the screen one at a time. Participants indicate if they remember seeing the word earlier or not.	Accuracy (%) and reaction time for the correct responses (ms)
Delayed picture recognition	All target pictures shown during Picture presentation plus an equal number of decoys are displayed on the screen one at a time. Participants indicate if they remember seeing the picture earlier or not.	Accuracy (%) and reaction time for the correct responses (ms)

Supplemental Table 2. Cognitive Demand Battery. Means and standard deviations (SD) are presented.

				Baseline		60 min p.d.		180 min p.d.		360 min p.d.	
		**Treatment**	**N**	**Mean**	**SD**	**Mean**	**SD**	**Mean**	**SD**	**Mean**	**SD**
	Repetition 1	Placebo	32	54.81	23.28	56.37	24.56	58.69	24.31	56.25	24.32
		Blueberry		53.84	24.38	58.03	24.58	60.56	25.16	56.53	23.68
		Apple		51.69	20.30	57.34	23.69	57.37	22.14	54.88	22.86
		Coffee berry		54.16	24.48	57.12	24.46	59.97	23.09	55.31	21.16
	Repetition 2	Placebo		51.91	25.16	54.38	25.81	56.19	24.49	53.34	24.19
		Blueberry		53.91	25.56	54.28	26.27	58.09	24.71	52.47	21.73
		Apple		51.53	22.14	50.69	22.37	56.16	22.33	50.75	21.60
Serial 3 subtractions		Coffee berry		51.78	22.63	55.00	23.86	56.81	23.32	52.65	21.94
Total (number)[1]	Repetition 3	Placebo		50.84	25.02	55.22	26.98	54.19	24.13	50.50	23.79
		Blueberry		52.06	25.60	53.47	25.52	55.62	25.37	51.22	25.14
		Apple		50.84	21.71	53.19	23.77	52.84	21.97	49.75	24.27
		Coffee berry		49.47	24.07	55.94	24.03	55.22	23.84	51.69	24.52
	Repetition 4	Placebo		51.06	25.47	52.16	23.35	53.84	25.18	52.81	24.48
		Blueberry		51.32	24.58	53.16	25.11	55.56	26.91	54.41	24.69
		Apple		48.59	21.23	53.44	24.40	54.84	23.15	52.48	23.14
		Coffee berry		52.31	24.70	56.78	25.50	57.06	24.49	52.41	22.15
Serial 3 subtractions	Repetition 1	Placebo	32	1.06	1.08	2.44	2.69	2.09	2.32	1.50	1.78

P.d., post dose; RVIP, rapid visual information processing

Errors (number)[1]		Blueberry		1.87	1.56	2.75	2.26	1.69	1.87	1.50	1.72
		Apple		1.44	1.63	2.19	2.55	2.13	2.20	1.56	1.74
		Coffee berry		1.53	1.83	2.78	2.59	1.31	1.55	1.66	1.62
	Repetition 2	Placebo		2.06	1.92	2.28	2.69	1.50	1.55	1.66	2.10
		Blueberry		1.66	1.94	2.69	2.49	2.16	1.90	2.31	2.46
		Apple		1.94	1.95	2.41	2.60	1.72	1.76	1.69	1.84
		Coffee berry		1.41	2.01	1.42	1.67	1.78	2.20	2.13	2.06
	Repetition 3	Placebo		2.66	2.59	2.28	2.67	2.13	2.52	2.41	1.85
		Blueberry		3.16	2.74	3.00	2.98	2.44	2.34	2.44	2.34
		Apple		2.09	2.33	2.84	2.84	2.22	2.30	2.19	2.32
		Coffee berry		2.19	1.97	2.23	2.08	2.09	1.91	2.16	2.33
	Repetition 4	Placebo		3.00	3.07	2.78	3.58	2.00	2.53	1.97	2.66
		Blueberry		2.23	2.17	2.66	2.31	1.47	1.78	2.06	2.27
		Apple		2.56	3.05	2.88	3.29	1.81	2.05	2.71	2.66
		Coffee berry		2.44	2.20	2.13	2.32	1.88	2.24	2.22	2.41
Serial 7 subtractions Total (number)[1]	Repetition 1	Placebo	32	33.28	17.45	36.59	18.56	36.81	19.18	36.31	18.31
		Blueberry		33.63	17.57	37.25	18.67	39.47	17.61	35.94	16.50
		Apple		31.81	14.81	34.84	14.43	35.78	15.98	33.47	15.30
		Coffee berry		32.97	16.19	36.47	16.90	37.59	18.94	36.22	17.96
	Repetition 2	Placebo		33.84	18.82	35.59	16.62	36.47	18.59	34.37	16.91

		Blueberry		34.19	18.18	35.38	18.58	36.97	17.16	34.66	16.88
		Apple		32.34	15.12	33.13	15.56	35.28	15.43	33.19	15.56
		Coffee berry		32.75	17.83	36.50	17.70	36.16	17.85	35.70	18.22
	Repetition 3	Placebo		33.75	18.73	35.44	17.73	36.03	18.53	34.44	15.85
		Blueberry		34.63	18.72	38.14	19.31	37.19	18.53	35.48	16.31
		Apple		32.31	15.23	31.69	14.96	35.09	15.83	31.70	15.74
		Coffee berry		31.78	16.34	36.37	18.13	34.91	17.07	34.61	17.41
	Repetition 4	Placebo		33.84	17.59	34.81	16.31	35.58	18.78	36.68	17.23
		Blueberry		35.28	17.72	35.34	17.38	36.41	17.83	36.28	16.72
		Apple		32.97	14.52	34.25	15.26	35.84	15.20	33.88	15.57
		Coffee berry		33.26	17.29	35.90	15.82	37.13	17.68	35.94	16.92
Serial 7 subtractions Errors (number)[1]	Repetition 1	Placebo	32	1.09	1.40	1.31	1.40	1.87	2.04	1.75	2.05
		Blueberry		1.66	1.58	2.34	2.15	1.88	2.08	2.00	2.02
		Apple		1.50	1.52	2.41	1.72	2.00	1.85	1.75	1.72
		Coffee berry		2.31	1.64	1.84	1.53	2.03	1.93	1.78	2.01
	Repetition 2	Placebo		2.16	2.22	2.41	1.98	1.72	1.94	1.97	1.99
		Blueberry		1.81	1.73	2.56	2.00	2.16	1.94	1.81	1.93
		Apple		1.91	1.92	2.28	2.58	1.87	1.54	2.39	2.29
		Coffee berry		2.63	1.79	2.59	2.38	2.16	2.52	2.23	1.92
	Repetition 3	Placebo		2.31	2.46	2.78	2.61	2.31	2.09	2.75	2.57

		Blueberry		2.75	2.16	3.03	2.99	2.34	2.44	2.39	1.76
		Apple		2.28	2.11	3.41	2.84	2.28	2.02	2.47	1.78
		Coffee berry		2.53	2.53	1.77	1.63	3.16	2.99	2.35	2.11
	Repetition 4	Placebo		3.23	2.53	2.29	1.79	2.45	2.58	2.13	1.73
		Blueberry		2.97	2.16	2.84	2.19	2.94	2.40	2.81	2.75
		Apple		2.65	1.80	3.09	2.32	2.28	2.05	2.50	2.14
		Coffee berry		2.42	2.03	2.61	2.60	2.06	2.11	2.34	1.94
	Repetition 1	Placebo	32	59.90	20.87	58.75	22.61	61.00	19.34	57.80	18.73
		Blueberry		62.12	20.40	59.62	21.50	60.10	22.43	60.96	22.10
		Apple		63.75	18.86	59.42	20.45	61.44	17.50	55.96	17.93
		Coffee berry		59.42	18.78	58.37	20.06	62.00	18.83	58.65	19.93
	Repetition 2	Placebo		57.69	22.16	53.08	22.53	53.60	20.76	52.12	18.01
		Blueberry		58.75	19.36	56.60	20.87	57.40	18.62	54.52	17.99
RVIP % correct[1]		Apple		60.48	17.89	55.19	19.01	54.13	17.82	50.31	16.78
		Coffee berry		56.50	18.87	58.70	20.20	55.90	18.85	55.21	20.48
	Repetition 3	Placebo		55.96	21.48	57.28	20.28	51.80	19.77	54.10	19.24
		Blueberry		58.02	21.14	56.35	20.53	54.10	20.85	51.83	20.68
		Apple		56.44	18.91	53.70	18.65	53.08	21.74	49.69	16.07
		Coffee berry		54.30	21.21	54.42	19.73	55.70	18.70	54.40	21.42
	Repetition 4	Placebo		53.75	17.88	51.44	22.32	57.39	16.45	53.37	20.77

		Blueberry		56.67	19.80	54.62	19.49	54.50	19.62	56.35	18.44
		Apple		53.94	19.15	54.81	20.37	52.80	19.41	49.69	17.90
		Coffee berry		52.20	19.30	55.00	19.01	54.23	21.42	52.60	18.06
	Repetition 1	Placebo	32	486.88	47.68	479.64	55.16	472.66	51.92	465.91	58.37
		Blueberry		482.91	66.20	472.78	51.47	477.25	52.52	464.06	46.93
		Apple		473.42	44.37	479.69	47.80	469.56	49.01	460.96	52.78
		Coffee berry		480.88	52.05	465.28	47.71	470.77	50.95	472.36	43.57
	Repetition 2	Placebo		493.34	60.76	478.94	57.74	482.99	50.82	477.15	47.02
		Blueberry		493.72	64.15	480.56	59.60	485.48	59.31	484.16	55.86
		Apple		483.72	48.04	488.25	51.20	473.92	51.60	475.41	59.16
RVIP		Coffee berry		491.04	49.84	468.65	50.80	471.05	54.40	474.65	62.98
Reaction time (ms)[1]	Repetition 3	Placebo		484.74	48.54	480.57	52.68	478.88	41.97	480.17	58.79
		Blueberry		486.35	65.05	490.00	60.71	484.73	55.29	484.21	47.54
		Apple		481.74	37.06	472.00	44.30	484.01	68.44	480.28	61.11
		Coffee berry		488.79	44.64	484.82	56.62	470.70	54.06	474.95	48.86
	Repetition 4	Placebo		485.05	47.17	474.20	53.79	477.93	43.68	471.38	51.75
		Blueberry		483.90	60.94	494.32	70.83	475.42	50.53	472.12	50.79
		Apple		484.56	45.74	485.81	48.27	473.53	53.41	489.63	59.71
		Coffee berry		483.58	55.61	481.46	53.89	474.97	57.56	479.34	61.93
Alertness[1]	Repetition 1	Placebo	31	45.09	17.22	45.31	17.47	45.31	17.26	43.81	18.22

		Blueberry		46.44	19.50	45.13	16.55	47.91	16.69	43.13	16.68
		Apple		49.00	19.47	44.94	18.19	50.81	18.46	43.91	16.42
		Coffee berry		48.50	18.78	45.06	19.43	49.66	18.62	47.34	19.33
	Repetition 2	Placebo		40.06	19.54	39.31	17.51	41.63	17.17	38.09	20.06
		Blueberry		45.38	22.01	42.72	18.87	40.97	19.90	36.31	18.12
		Apple		44.53	21.41	41.47	17.81	46.56	20.37	39.91	18.22
		Coffee berry		42.66	19.26	48.25	19.58	45.81	20.21	38.28	19.78
	Repetition 3	Placebo		35.63	20.31	36.16	20.22	37.69	20.57	37.12	22.22
		Blueberry		42.03	21.80	39.56	18.88	37.06	22.33	31.37	17.45
		Apple		40.84	21.31	37.50	17.80	43.97	21.13	36.13	18.35
		Coffee berry		37.47	20.05	45.53	21.79	41.22	20.50	34.38	20.63
	Repetition 4	Placebo		37.19	21.57	36.53	19.87	36.19	20.86	36.53	23.29
		Blueberry		39.50	21.28	36.25	21.07	41.81	24.35	32.47	17.01
		Apple		38.66	20.14	37.88	19.61	45.28	21.95	38.81	18.11
		Coffee berry		35.97	21.09	45.25	23.03	42.28	21.94	35.56	21.03
	Repetition 1	Placebo	31	45.16	19.52	47.75	17.79	49.00	16.43	52.13	18.04
		Blueberry		46.78	18.06	48.53	17.21	47.22	18.55	52.00	17.30
Mental fatigue[1]		Apple		41.53	18.75	46.66	19.21	42.50	18.08	50.34	17.36
		Coffee berry		42.31	18.92	44.53	20.78	43.81	19.68	49.66	20.90
	Repetition 2	Placebo		49.06	20.76	54.97	17.67	55.00	16.68	59.44	20.95

	Blueberry	49.66	20.19	56.75	18.48	53.47	20.91	61.84	16.83
	Apple	43.03	19.99	50.66	18.81	45.97	21.22	53.06	20.66
	Coffee berry	48.69	20.84	46.72	21.93	51.81	22.11	60.31	20.59
Repetition 3	Placebo	51.47	23.61	56.59	19.17	57.94	19.27	61.31	21.96
	Blueberry	53.81	22.71	55.44	19.87	53.84	24.28	64.31	18.17
	Apple	49.59	21.83	54.75	19.19	46.19	22.96	60.47	18.91
	Coffee berry	55.31	22.47	47.41	21.77	53.72	23.42	62.03	19.50
Repetition 4	Placebo	56.03	23.48	60.81	19.55	58.97	22.46	61.13	22.71
	Blueberry	57.34	23.04	56.41	20.43	56.37	24.40	64.56	19.29
	Apple	51.59	21.17	57.75	19.70	50.78	23.65	59.69	18.42
	Coffee berry	57.00	22.78	48.78	24.65	55.50	23.20	66.06	19.59

[1]variable was log-transformed in the analysis

Supplemental Table 3. Cognitive and mood assessments. Means and standard deviations (SD) are presented.

				Baseline		60 min p.d.		180 min p.d.		360 min p.d.	
		Treatment	N	Mean	SD	Mean	SD	Mean	SD	Mean	SD
Delayed word recognition	% correct[1]	Placebo	32	80.10	7.64	77.71	8.31	75.70	10.72	74.95	9.73
		Blueberry		80.31	11.24	77.82	9.44	75.67	7.98	74.37	10.66
		Apple		78.49	11.89	77.63	9.78	75.40	9.55	74.71	7.48
		Coffee berry		78.96	10.69	75.00	8.76	76.15	9.77	76.04	9.71
	Reaction time(ms)[1]	Placebo		821.29	217.65	856.52	325.56	802.91	187.30	834.89	231.94
		Blueberry		828.35	228.61	799.70	181.17	824.22	210.70	797.61	187.48
		Apple		820.19	180.93	856.92	228.97	811.84	171.28	861.58	227.58
		Coffee berry		830.41	246.72	895.51	281.70	842.07	292.03	851.47	234.02
Delayed picture recognition	% correct[1]	Placebo	32	91.46	9.20	87.19	11.71	88.28	11.28	88.75	10.36
		Blueberry		93.44	6.90	91.94	7.14	88.85	9.56	88.33	10.58
		Apple		92.50	6.67	90.21	10.33	89.48	10.30	90.73	8.54
		Coffee berry		91.25	8.87	88.44	9.35	90.83	11.45	88.65	9.42
	Reaction time(ms)[1]	Placebo		788.68	165.87	826.08	176.89	800.33	167.01	778.21	154.62
		Blueberry		796.05	148.56	808.22	168.61	795.05	152.34	779.40	176.44
		Apple		809.08	230.20	827.86	197.94	797.38	195.05	814.68	160.90

		Coffee berry		789.60	140.08	828.61	198.99	783.01	191.35	807.51	207.78
Delayed word recall	Correct (number)[1]	Placebo	32	5.95	2.57	4.37	2.81	4.20	2.30	3.18	2.55
		Blueberry		6.03	3.02	4.27	3.08	3.64	2.48	3.40	2.63
		Apple		5.40	3.05	3.37	2.20	3.61	2.32	2.48	2.01
		Coffee berry		5.48	2.62	3.45	2.81	3.91	2.27	2.84	2.54
	Errors (number)[1]	Placebo		0.91	0.86	1.19	1.57	0.93	1.05	1.53	1.61
		Blueberry		0.75	1.02	1.19	1.47	1.34	1.79	1.39	1.65
		Apple		0.77	0.76	1.28	1.25	1.37	1.22	1.48	1.15
		Coffee berry		0.81	1.20	1.71	1.55	1.50	1.37	1.84	1.74
Immediate word recall	Correct (number)[1]	Placebo		8.31	2.48	8.28	2.30	8.38	2.47	7.85	1.94
		Blueberry		8.27	2.71	8.26	2.25	7.84	2.01	7.90	2.42
		Apple		8.48	2.43	7.67	1.79	8.10	2.09	7.58	1.76
		Coffee berry		8.20	2.30	7.73	2.40	8.59	2.17	8.00	2.55
	Errors (number)[1]	Placebo		0.56	0.67	0.25	0.44	0.37	0.67	0.47	0.63
		Blueberry		0.53	0.80	0.42	0.67	0.34	0.55	0.65	0.71
		Apple		0.44	0.67	0.50	0.76	0.33	0.48	0.48	0.81

		Coffee berry		0.28	0.73	0.61	0.80	0.44	0.80	0.66	0.79
		Placebo	32	95.78	5.10	95.62	5.55	95.94	3.78	96.61	3.84
	% correct[1]	Blueberry		97.45	2.96	95.83	2.74	97.29	3.51	96.51	3.12
		Apple		96.93	2.28	96.41	3.06	95.68	3.69	96.09	3.30
		Coffee berry		96.30	3.73	96.04	4.10	96.30	3.90	96.15	3.77
Stroop		Placebo		675.02	250.93	663.67	198.45	666.48	174.08	647.50	202.62
	Reaction time(ms)[1]	Blueberry		683.12	207.33	675.14	180.22	626.14	115.77	629.25	158.62
		Apple		678.75	242.57	680.69	253.56	654.97	181.14	643.05	164.09
		Coffee berry		682.19	287.92	662.12	305.57	654.69	209.03	660.34	230.23
		Placebo	32	2461.22	1190.35	2296.38	1042.40	2363.31	1148.75	2243.84	982.42
	Thinking time	Blueberry		2643.69	1261.78	2619.38	1345.70	2437.22	1299.43	2275.66	1096.69
Peg and Ball	(ms)[1]	Apple		2542.16	995.02	2375.87	1028.06	2431.25	1019.38	2300.53	980.46
		Coffee berry		2715.31	1080.04	2675.97	1116.68	2529.31	1355.43	2235.13	848.62
		Placebo		6908.50	1213.11	6682.09	1358.81	6297.91	968.60	6260.09	1135.46

	Completion time	Blueberry		7115.94	1585.61	6949.78	1388.45	6197.53	1101.62	6056.38	1094.72
	(ms)[1]	Apple		7050.37	1231.54	6734.44	1243.48	6444.44	1042.15	6410.87	1169.19
		Coffee berry		7152.78	1498.99	7075.22	1632.77	6483.03	1234.98	6320.94	1008.21
		Placebo		2.03	2.16	2.66	2.60	3.13	3.15	2.37	2.65
	Errors	Blueberry		2.81	2.40	3.41	2.23	2.00	2.26	2.63	2.57
	(number)[1]	Apple		2.25	2.37	2.97	2.72	2.59	2.35	2.34	2.36
		Coffee berry		2.50	2.82	4.09	3.64	2.78	3.48	2.77	2.91
		Placebo	31	45.21	13.35	42.94	14.20	46.01	15.54	46.17	15.56
		Blueberry		44.35	15.41	43.03	14.63	46.57	15.70	41.64	12.09
	Alert	Apple		46.89	13.51	45.62	12.68	49.80	13.32	44.01	11.21
		Coffee berry		44.24	11.55	49.27	14.30	48.48	15.15	43.79	14.39
Bond-Lader VAS		Placebo	31	61.36	9.76	59.48	11.64	59.73	12.22	60.03	15.01
		Blueberry		61.20	13.42	62.63	13.90	58.92	15.51	58.09	15.51
	Content[1]	Apple		60.20	13.41	60.27	12.82	58.56	13.22	58.08	11.26
		Coffee berry		58.89	10.23	59.39	11.99	58.34	12.33	57.20	14.21
	Calm[1]	Placebo	31	55.85	12.90	55.74	13.43	54.94	14.27	57.68	13.54

		Blueberry		57.11	12.77	58.34	11.07	58.86	12.79	56.99	10.62
		Apple		57.90	13.00	59.21	10.21	60.11	11.81	59.34	13.42
		Coffee berry		56.98	8.65	59.38	11.17	58.67	11.61	57.66	11.80
VAS	Clear mind[1]	Placebo	32	42.59	16.99	43.31	16.66	42.88	17.91	46.72	18.98
		Blueberry		41.59	16.79	42.19	17.18	44.31	16.97	38.47	15.34
		Apple		45.41	16.68	43.66	16.66	45.41	16.12	41.28	17.71
		Coffee berry		41.81	16.51	44.69	16.52	41.28	17.89	39.47	17.22
	Focused	Placebo	32	39.72	16.25	40.88	16.86	44.16	19.72	43.47	18.26
		Blueberry		44.97	17.42	41.56	15.50	45.81	19.14	38.16	15.49
		Apple		46.88	15.93	44.94	16.58	49.13	17.16	42.94	15.59
		Coffee berry		40.78	13.68	47.03	16.29	44.78	18.40	38.59	16.49
	Problem solving[1]	Placebo	32	46.66	16.11	46.72	16.08	47.66	20.25	47.44	20.30
		Blueberry		46.13	17.52	42.75	19.05	48.31	19.30	42.06	15.69
		Apple		46.63	16.27	44.00	16.14	51.19	17.39	44.66	17.09
		Coffee berry		44.31	15.78	49.50	17.84	48.09	19.10	45.84	18.15
	Productive	Placebo	32	42.34	15.10	42.44	17.46	44.56	19.56	42.84	18.13
		Blueberry		41.87	16.53	41.72	17.18	43.00	18.85	37.00	16.89

	Apple	45.72	16.24	43.59	15.66	47.72	17.68	44.34	16.27
	Coffee berry	41.25	16.63	47.28	17.75	48.44	16.18	43.00	17.48

P.d., post dose; VAS, visual analogue scales

[1]variable was log-transformed in the analysis

Supplemental Table 4. Profile of Mood States. Mean and standard deviations (SD) are presented.

			Baseline		60 min p.d.		180 min p.d.		360 min p.d.	
**Subscale**	**Treatment**	**N**	**Mean**	**SD**	**Mean**	**SD**	**Mean**	**SD**	**Mean**	**SD**
	Placebo	32	1.94	2.83	2.81	4.55	3.00	4.64	2.68	5.26
Anger/Hostility[1]	Blueberry		3.13	5.68	2.68	4.95	2.52	5.60	2.90	5.88
	Apple		3.10	5.44	2.44	4.59	1.84	3.73	2.97	6.35
	Coffee berry		2.44	4.60	2.47	5.26	2.47	4.75	2.97	6.34
	Placebo	32	11.28	6.30	12.03	5.88	11.56	5.96	11.71	5.38
Confusion/Bewilderment[1]	Blueberry		12.61	7.54	12.55	7.10	11.13	6.46	12.10	6.68
	Apple		11.77	6.81	11.56	6.40	11.66	6.97	12.39	6.41
	Coffee berry		11.81	4.97	9.47	5.28	10.38	5.80	11.22	5.64
	Placebo	32	3.53	5.05	4.59	6.61	4.13	5.74	4.13	6.52
Depression/Dejection[1]	Blueberry		5.48	8.35	4.52	7.87	4.32	7.87	4.68	8.32
	Apple		3.94	8.87	4.03	6.98	3.59	7.51	3.77	6.94
	Coffee berry		3.09	5.59	3.47	6.57	3.09	5.94	3.88	7.22
	Placebo	32	8.66	6.02	8.78	5.00	8.34	5.45	9.77	5.18
Fatigue/Inertia[1]	Blueberry		8.81	6.36	9.06	6.26	7.00	5.94	10.10	6.62
	Apple		8.29	6.56	7.63	5.61	7.78	6.20	9.77	5.92
	Coffee berry		8.37	5.54	6.38	5.76	7.06	6.04	9.03	6.30
Tension/Anxiety[1]	Placebo	32	6.22	6.33	7.06	5.67	6.94	5.49	6.84	5.36

	Blueberry		7.19	6.77	7.03	6.40	7.35	7.08	7.90	8.17
	Apple		6.97	7.03	5.53	5.10	6.06	6.06	7.19	6.22
	Coffee berry		7.03	6.46	6.09	5.99	6.50	5.02	7.22	7.16
	Placebo	32	9.81	5.99	7.56	5.87	9.53	6.43	8.81	6.51
Vigour/Activity[1]	Blueberry		9.00	6.00	8.19	5.79	10.65	6.92	7.81	5.29
	Apple		9.00	5.81	8.63	6.36	9.63	5.24	8.13	5.51
	Coffee berry		8.69	5.13	11.12	7.92	10.13	7.62	9.81	6.83
	Placebo	32	21.81	25.86	27.72	24.62	24.44	26.29	26.32	23.01
Total Mood Disturbance (TMD)[1]	Blueberry		28.23	32.41	27.65	28.33	21.68	30.32	29.87	30.97
	Apple		25.06	31.32	22.56	25.48	21.31	27.37	27.97	28.12
	Coffee berry		24.06	22.29	16.75	26.63	19.37	25.24	24.50	28.97

[1]variable was log-transformed in the analysis

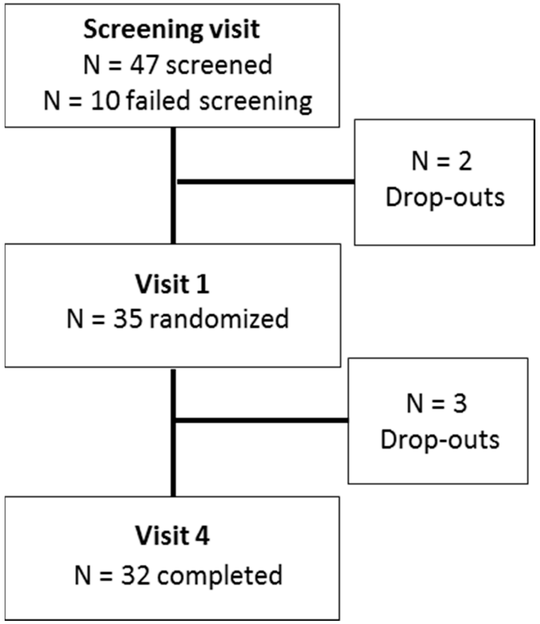

Supplemental Figure 1. Flow diagram of disposition of subjects throughout the study

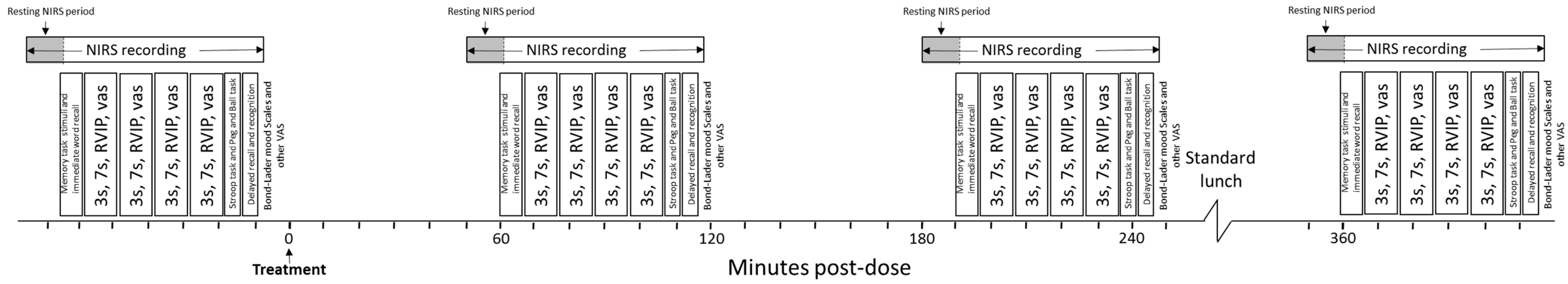

Supplemental Figure 2. Schedule of each testing visit. Participants arrived at 8am or 9am. They completed one baseline cognitive assessment, then sat quietly for 5 minutes before commencing a 10 minute pre-dose resting baseline Near Infrared Spectroscopy (NIRS) assessment. They then took their day's treatment and 50 minutes post-dose underwent a 10-minute NIRS assessment while resting, which continued while they completed their first post-dose cognitive assessment (starting at 60 minutes post-dose). This post-dose assessment was repeated commencing at 170 minutes and 350 minutes post-dose.
